# Single-cell RNA sequencing of immune cells in patients with acute gout

**DOI:** 10.1038/s41598-022-25871-2

**Published:** 2022-12-22

**Authors:** Jan-Gowth Chang, Siang-Jyun Tu, Chung-Ming Huang, Yu-Chia Chen, Hui-Shan Chiang, Ya-Ting Lee, Ju-Chen Yen, Chia-Li Lin, Chin-Chun Chung, Ta-Chih Liu, Ya-Sian Chang

**Affiliations:** 1 Center for Precision Medicine, China Medical University Hospital, 2 Yuh-Der Road, Taichung, 404 Taiwan; 2 Epigenome Research Center, China Medical University Hospital, 404 Taichung, Taiwan; 3 School of Medicine, China Medical University, Taichung, Taiwan; 4 Department of Bioinformatics and Medical Engineering, Asia University, Taichung, Taiwan; 5 Department of Laboratory Medicine, China Medical University Hospital, Taichung, Taiwan; 6 Division of Immunology and Rheumatology, Department of Internal Medicine, China Medical University Hospital, Taichung, Taiwan; 7 Department of Hematology-Oncology, Chang Bing Show Chwan Memorial Hospital, 6 Lugong Road, Changhua, 505 Taiwan

**Keywords:** Genetics, Molecular biology

## Abstract

Cell subpopulations in the blood and joint fluid of patients with gout are poorly understood. Single-cell RNA sequencing and bioinformatic tools were used to identify cell subsets and their gene signatures in blood and synovial fluid (SF) cells, determine their relationships, characterize the diversity, and evaluate interactions among specific cell types. We identified 34 subpopulations (5 types of B cells, 16 types of T and natural killer cells, 9 types of monocytes, and 4 other cell types) in the blood of five healthy subjects and seven patients with acute gouty, and the SF of three patients with acute gout. We found that naïve CD4 T cells and classical monocytes cell populations were enriched in patients with gout, whereas plasmacytoid dendritic cells and intermediate monocytes were more abundant in healthy subjects. SF was enriched in Th1/Th17 cells, effector memory CD8 T cells, mucosal-associated invariant T cells, and macrophages. Subclusters of these cell subpopulations showed different compositions between healthy subjects and those with acute gout, according to blood and SF samples. At the cellular level, the inflammation score of a subpopulation or subcluster was highest in SF, following by the blood of acute gout patients and healthy person, whereas energy score showed the opposite trend. We also detected specific cell–cell interactions for interleukin-1, tumor necrosis factor-α, and transforming growth factor-β1 expression in the cells of patients with acute gout. Our study reveals cellular and molecular insights on inflammatory responses to hyperuricemia or uric crystal and may provide therapeutic guidance to improve treatments for gout.

## Introduction

Gout is a crystal-associated joint disease caused by the precipitation of monosodium urate (MSU) resulting from hyperuricemia, which is in turn caused by disordered purine metabolism and/or a decrease in uric acid excretion^1,2^. Hyperuricemia is the most important factor for the development of gout, and serum urate (SU) concentrations are significantly correlated with gout. Hyperuricemia alone is insufficient for gout induction; over a 15-year period, only 9% of patients with hyperuricemia (SU > 7.0–8.9 mg/dL) experience a gout flare, and even at SU levels above 10 mg/dL, only 50% of patients develop clinically evident gout^3^. Many patients with hyperuricemia do not develop clinical gout, which suggests that factors other than MSU crystal deposition are important to gout development^4^. Worldwide gout incidence increases yearly, influenced by economic booms that have resulted in human environmental, lifestyle, and dietary changes^5,6^. Gout has been variably classified as a metabolic disorder and an autoinflammatory disease^3,7,8^. Innate inflammatory pathways play an important role in the pathogenesis of gout according to in vitro, ex vivo, and animal studies. Gout development is mediated by interactions between MSU precipitation; the activity of immune cells including macrophages, monocytes, lymphocytes, and neutrophils; and their secretions such as tumor necrosis factor-α (TNF-α), interleukin (IL)-1β, IL-8, IL-10, IL-17, IL-37, NACHT, LRR, and PYD domain-containing protein 3 inflammasome, which induce a series of cascade inflammatory amplification reactions^9–11^. Because most studies of gout development have been conducted using cultured or bulk cell systems, the detailed compositions and interactions of these cell types are still unknown, and biomarkers for the effective prediction of gout and the cellular heterogeneity leading to gout progression remain to be identified.

Single-cell RNA sequencing (scRNA-seq) is a powerful method for investigating variation among cells using transcriptomic markers. scRNA-seq has been performed to study various diseases including different types of inflammatory arthritis, but has not yet been applied to gout studies^12,13^. To explore the molecular and cellular involvement of cell subpopulations in the blood and synovial fluid (SF) of patients with gout, we performed scRNA-seq and identified the gene signatures of various cell subsets and their diversity in specific cell types in the blood and SF of patients with acute gout. Finally, we investigated the influence of cellular heterogeneity and identified potential therapeutic targets.

## Methods

### Patient recruitment and ethics

Peripheral blood and SF were obtained from patients who fulfilled the 2010 American College of Rheumatology/European League Against Rheumatism (ACR/EULAR) classification criteria for gouty arthritis. We collected basic patient information including age, uric acid level, glomerular filtration rate, and disease duration. Informed consent was obtained from all participants, and the study was approved by the Research Ethics Committee of China Medical University Hospital, Taiwan (CMUH108-REC2–051).

### ScRNA-seq analysis

Mononuclear cells were isolated from peripheral blood using Ficoll-Paque gradient centrifugation. Total cells were collected from SF. Single-cell 3′ gene expression libraries of mononuclear and SF cells were prepared using the Chromium Single Cell 3’v2 Library kit (10× Genomics; Pleasanton, CA, USA), according to the manufacturer’s protocol. All libraries were sequenced on the Illumina platform (NovaSeq 6000; Illumina, San Diego, CA, USA). Raw data were progressed and aligned to the GRCh38–3.0.0 human transcriptome using the CellRanger v3.1.0 or v6.0.2 software (10× Genomics). Using the *Seurat* v4.0.4 package^13^ of the R software, cell data were collected under the following criteria: 500–4500 genes detected, < 30,000 unique molecular identifiers (UMI), < 15% reads from mitochondria, and no potential doublets detected or predicted by *Seurat* Scrublet v0.2.2 validation^14^ with the default parameters. File type conversion was performed using the Scanpy v1.8.1^15^ and SeuratDisk v0.0.0.9015 tools.

### Integration of the scRNA-seq dataset

We integrated all scRNA-seq datasets using *Seurat*. In each dataset, we estimated cell cycle stages according to function using the CellCycleScoring function, and then performed SCTransform analysis. The regression variables included cell cycle stage, mitochondria read abundance, gene number, and UMI count. The SelectIntegrationFeatures function was used to find the 3,000 most highly variable genes for downstream principal component analysis (PCA). For large dataset integration, we performed a reference-based integration workflow with robust PCA (rPCA). Five health control datasets were selected to form a reference dataset.

### Main cell type annotation

We performed uniform manifold approximation and projection (UMAP) with the top 50 PCs obtained from PCA and shared nearest neighbor (SNN) analysis. To identify the main cell types, we applied the automated cell type annotation tool SingleR v1.4.1^16^. The transcriptome expression of each cell was compared to built-in reference datasets in the celldex v1.0.0 index^16^. Each cell was labeled with specific nomenclature and a cell ontology term from the reference dataset. First, we selected cell type labels using the MonacoImmuneData function. Then, we selected the labels Macrophages M1 and Macrophages M2 using the BlueprintEncodeData function. Finally, Macrophages (CL: 0000235), Lung Macro (CL: 0000583), INF-Macro (CL: 0000863) and Megakaryocyte (CL: 0000556) types were identified according to cell ontology terms.

### Main cell type clustering and gene marker identification

We extracted cell types from the integrated dataset and performed PCA, UMAP, and SNN following the same parameters used in the previous analysis. The resolution ranged from 0.1 to 0.8 and the number of clusters ranged from 3 to 12. To analyze the expression of cluster-related genes, we applied the FindAllMarkers function in *Seurat* to each selected cluster of each selected main cell type using the default settings.

### Inflammation score (IS) and energy score (ES) evaluation

We obtained inflammatory genes from the Molecular Signatures Database v7.5 HALLMARK_INFLAMMATORY_RESPONSE web page (http://www.gsea-msigdb.org/gsea/msigdb)^17^. The energy gene set contained 11 metabolism-related pathways according to the Reactome pathway database (https://reactome.org)^18^. We used the AddModuleScore function in Seurat to calculate IS and ES.

### Cell–cell interactions

The R package *CellChat* v1.1.3^19^ was used to infer communication probabilities for pro-inflammation (IL-1 and TNF-α) and anti-inflammation (transforming growth factor-β1 [TGF-β1]) signals among all cell groups in the peripheral blood scRNA-seq datasets.

### Statistical analysis

The main cell types and clustering relative abundance are expressed as percentages, and IS and ES are unitless. Continuous data were compared using Wilcoxon rank sum tests using the R package *ggpubr* v0.4.0 (https://rpkgs.datanovia.com/ggpubr). Differences were considered significant at *P* < 0.05.

### Ethics approval and consent to participate

Informed consents were obtained from all participants, and the study was approved by the Research Ethics Committee of China Medical University Hospital, Taiwan (CMUH108-REC2-051). Both the Declaration of Helsinki and the Good Clinical Practice Guidelines were followed and informed consent granted by all participants.

## Results

### Clinical conditions of patients with gout

The clinical conditions of seven patients with acute gout are shown in Table S1. Among these patients, only Case 07 was a new case, with the first attack lasting for 19 days; all others had a disease history of 1 to > 20 years, with attacks lasting 1–14 days. Cases 03 and 07 were heavy drinkers, and Case 05 drink occasionally; the other subjects did not drink.

### Landscape of immune cells from healthy subjects and patients with gout

**Figure 1:**
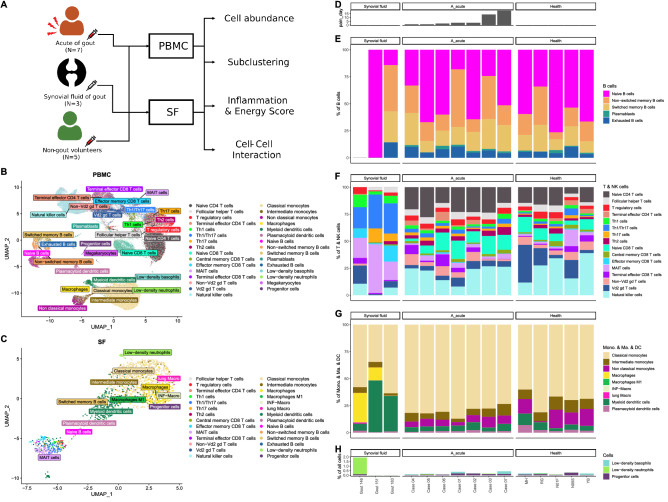
Immunological landscape of immune cells from healthy subjects and patients with gout. (**A**) Study design. Immune cells extracted from (**B**) peripheral blood mononuclear cells (PBMCs) and (**C**) synovial fluid (SF) are presented in uniform manifold approximation and projection (UMAP) coordinates. (**D**) Bar chart of pain duration in patients with gout. Relative abundance of various cell subpopulations: (**E**) B cells; (**F**) T and NK cells; (**G**) a monocyte series including monocytes (Mono.), macrophages (Ma.), and dendritic cells (DCs); and (**H**) megakaryocytes, progenitor cells, low-density neutrophils, and low-density basophils.

To investigate immune cell phenotype heterogeneity in healthy subjects and patients with gout, we performed scRNA-seq analysis of peripheral blood mononuclear cells (PBMCs) isolated from healthy controls (n = 5) and patients with gout (n = 7), and SF cells from patients with acute gout (n = 3) (Fig. 1A). After data preprocessing and quality control, we obtained single-cell transcriptomes for 79,465 and 47,495 immune cells from PBMCs of subjects with and without gout, respectively, and 1984 immune cells from SF. To identify cell subpopulations in PBMCs and SF from gout patients and PBMCs from healthy controls, we performed SCTransform normalization to regress out UMI counts, gene numbers, mitochondrial read percentages, and cell cycle stages in all PBMCs and SF cells, and then performed rPCA on the integrated normalized dataset using *Seurat*. After SingleR annotation, 31 immune cell subpopulations were predicted from blood samples including B cells, T cells, monocytes, macrophages, dendritic cells (DCs), natural killer (NK) cells, megakaryocytes, granulocytes, and progenitors (Fig. 1B), and 28 immune cell subpopulations were predicted from SF (Fig. 1C).

We also analyzed pain duration among the subjects (Fig. 1D). We found high diversity of immune cell subpopulations among patients with gout, and similar results were obtained in healthy subjects. The B cell subpopulations included naïve B, non-switched memory B, switched memory B, plasmablasts, and exhausted B cells, with no useful pattern for differentiating patients with gout from healthy subjects (Fig. 1E).

We used 16 subpopulations for T and NK cell analysis; the results showed high T cell diversity in the blood of patients with gout and healthy subjects, and immune cell population patterns were influenced by the clinical course of the gout patients, with less cell diversity among gout patients experiencing similar pain duration. For example, patients with longer joint pain duration appeared to have fewer terminal effector CD4, terminal effector CD8, and non-Vd2 gd T cells, but more Th2 cells. No such pattern was observed among healthy subjects. SF samples contained more Th1, Th1/Th17, effector memory CD8, and mucosal-associated invariant T (MAIT) cells than blood samples from patients with acute gout and healthy subjects (Fig. 1F).

Among the nine monocyte cell populations detected, more intermediate monocytes and plasmacytoid DCs (pDCs) were found in healthy subjects than in patients with acute gout, whereas the latter group had more classical monocytes in the blood. Macrophages and myeloid DCs (mDCs) were the major cells in SF (Fig. 1G).

Among other cell types, we detected four cell population types including megakaryocytes, progenitor cells, low-density neutrophils, and low-density basophils. The lack of low-density neutrophils in the blood may have resulted from the experimental procedure used for mononuclear cell selection. Cell populations in this were similar between patients with acute gout and healthy subjects (Fig. 1H).

### Cell populations in PBMCs and SF from acute gout patients and healthy controls

**Figure 2:**
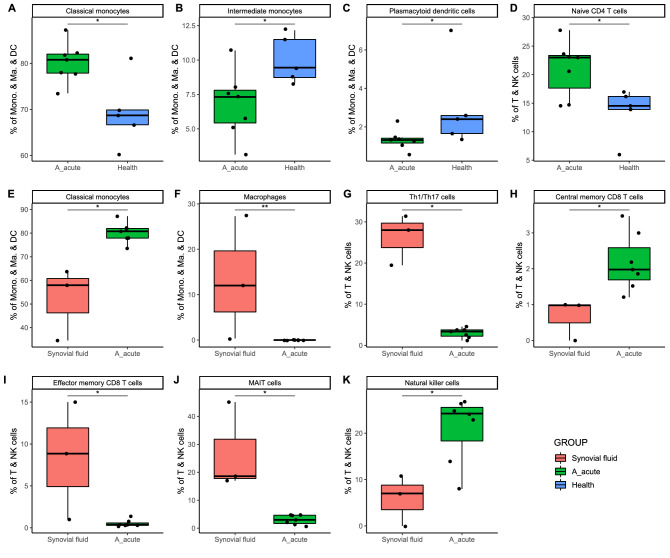
Relative abundance of cell subpopulations between PBMCs from acute gout patients and either PBMCs from healthy subjects or SF. (**A**–**D**) Relative abundance of cell subpopulations in PBMCs from acute gout and healthy subjects. (**E**–**K**) Relative abundance of cell subpopulations compared between PBMCs from patients with acute gout and SF samples. The cell subpopulations examined in this comparison were (**A**–**F**) monocyte series and (**G**–**K**) T and natural killer (NK) cells. *P < 0.05; **P < 0.01.

To evaluate differences in the cell populations PBMCs and SFs, we performed Wilcoxon rank sum tests to determine the relative abundance of each cell type. The results showed that naïve CD4 and classical monocyte cell populations were enriched in patients with acute gout, and pDCs and intermediate monocytes were more abundant in healthy subjects. Th1/Th17, effector memory CD8, MAIT, and macrophage cells were the major cells in SF (Fig. 2).

### Cell subclusters in PBMCs and SF in patients with acute gout and healthy controls

**Figure 3:**
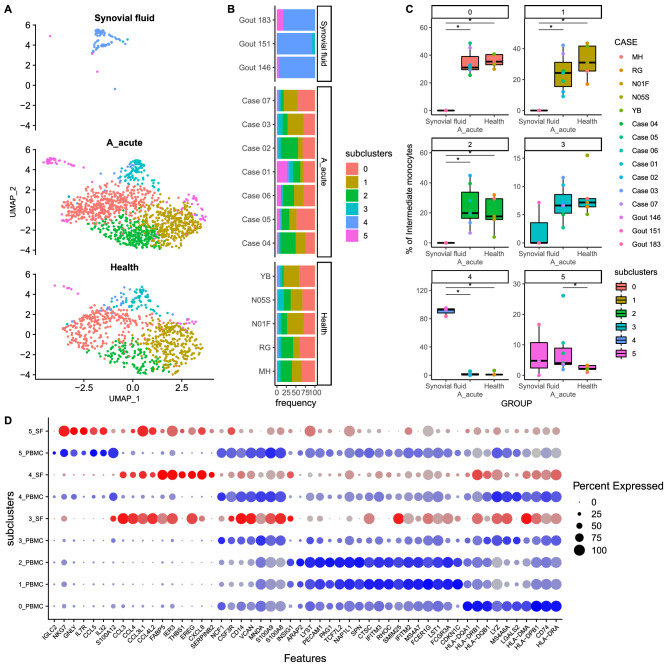
Intermediate monocyte subclustering results for (**A**,**B**) SF and PBMCs from acute gout and healthy subject groups are shown on UMAP coordinates. Stacked bar plot shows relative abundances. (**C**) Boxplots of subcluster relative abundance between SF samples and PBMCs of acute gout and healthy subject groups (*P < 0.05). (**D**) Dot plot of gene expression profiles of the top 10 marker genes in each subcluster.

We further subclustered the subpopulation of immune cell types detected in PBMCs and SF. The results indicated increased levels of subcluster 5 intermediate monocytes (Fig. 3), subcluster 1 MAIT cells (Fig. S1), and subcluster 1 regulatory T cells (Fig. S2) in the blood of patients with gout compared to healthy subjects. Gene panel analysis results obtained through UMAP analysis showing the distribution of intermediate monocyte cell subclusters in PBMCs and SF of acute gout and control groups are presented in Fig. 3A. Subclusters 0, 1, and 2 were dominant in patients with acute gout and healthy controls, subcluster 4 was dominant in SF, and subcluster 5 was more prevalent in acute gout patients than in healthy subjects (Fig. 3B,C). The gene panel used for subclustering is shown in Fig. 3D.

More MAIT cells were represented by subclusters 0 and 2 in patients with acute gout than SF, whereas SF contained more subcluster 3 MAIT cells, and acute patients with gout had dramatically more subcluster 1 cells than healthy subjects (Fig. S1). Among regulatory T cells, subclusters 1, 2, 3, and 4 were dominant in the blood of acute gout patients, whereas subcluster 0 was dominant in SF, and only subcluster 1 had significantly different blood levels between acute gout patients and healthy subjects (Fig. S2). Among Th1/Th17 cells, subcluster 0 was dominant in SF, and no significant differences among subclusters were detected in the blood of patients with acute gout and healthy subjects (Fig. S3). Only a few NK cells were detected in SF, and most NK cells were classified as subcluster 10, with no significant difference in blood samples between patients with acute gout and healthy subjects (Fig. S4). Three macrophage subclusters were detected in SF, and two subjects showed dominant subcluster 2 levels, with higher MHCII expression (Fig. S5). Too few macrophages were detected in the PBMCs of gout patients and healthy controls to evaluate significant differences.

### IS and ES of cell subpopulations and subclusters in blood and SF from acute gout and healthy subject groups

**Figure 4:**
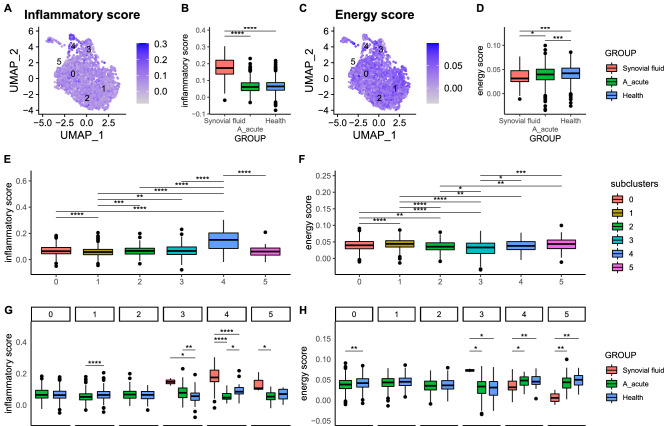
Inflammation score (IS) and energy score (ES) of intermediate monocytes. (**A**–**D**) Intermediate monocyte IS and ES values are shown on UMAP coordinates. Boxplots show scores among SF (red) and PBMC samples for acute gout (green) and health (blue) subject groups. (**E**,**F**) Boxplots of ISs and ESs among subclusters according to cluster labels. (**G**,**H**) ISs and ESs among SF (red) and PBMC samples for acute gout (green) and healthy (blue) subject groups for each subcluster. (*P < 0.05; **P < 0.01; ***P < 0.001; ****P < 0.0001).

We analyzed IS and ES in cell subpopulations and subclusters of patients with acute gout and healthy subjects using transcriptomic data. There were significant differences in IS and ESs within and among cell types, subpopulations, and subject groups for both blood and SF samples (Fig. 4). For example, the IS of intermediate monocytes was highest in SF, followed by PBMCs of gout and healthy control subjects, whereas ES showed the opposite trend. The IS of intermediate monocyte subclusters 4 and 5 were highest in SF, followed by the acute gout and healthy subject groups, whereas ES showed the opposite trend.

### Immune cell interactions in blood samples from patients with acute gout

**Figure 5:**
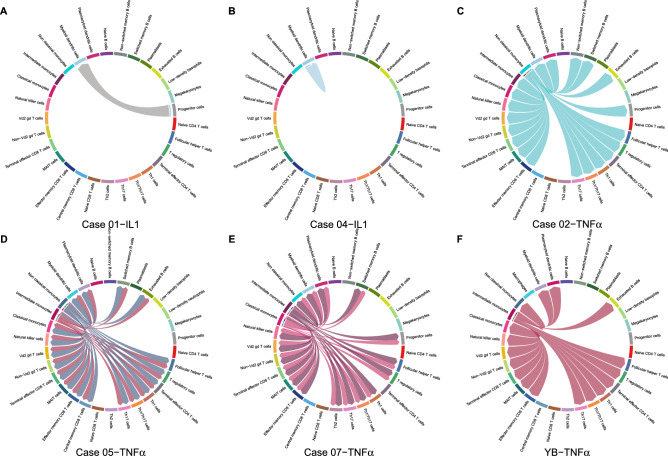
Cell–cell interaction signaling of IL-1 and TNF-α. (**A**,**B**) IL-1 signaling for Cases 01 and 04. (**C**–**F**) TNF-α signaling for Cases 02, 05, 07, and hyperuricemia (uric acid = 7.2) health control YB. Arrows from source cells to target cells indicate inferred signaling ligand secretion and reception.

Cytokine levels often increase in response to hyperuricemia or urate crystal stimulation, resulting in various interactions among immune cells in blood and synovial cells, eventually causing gout. We used the CellChat algorithm to explore these interactions. The results indicated mDC autointeraction or progenitor cell interaction in two patients with gout in response to IL-1-related stimulation (Fig. 5).

Numerous cell–cell interactions occurred following TNF-α stimulation, including various monocyte subtype interactions with immune cells; for example, non-classical monocytes interacted with different types of T cells, B cells, and monocytes in Case 02, and similar results were observed in two other patients with acute gout and one healthy control showing hyperuricemia (Fig. 5).

**Figure 6:**
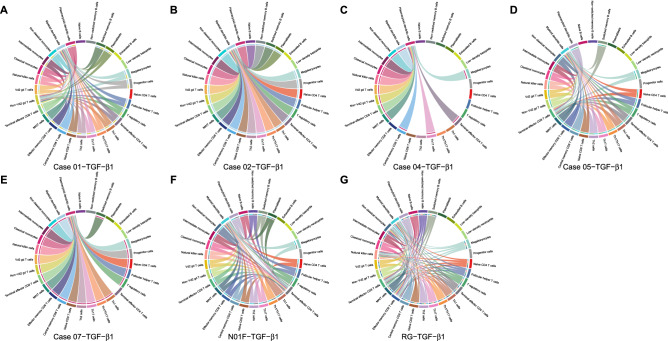
Cell–cell interaction signaling of TGF-β1. (**A**–**G**) TGF-β1 signaling for Cases 01, 02, 04, 05, 07, health control N01F, and RG. Arrows from source cells to target cells indicate inferred signaling ligand secretion and reception.

Following TGF-β1 stimulation, this cytokine was secreted by many immune cells to react with pDCs and mDCs in five patients with acute gout and two control subjects (Fig. 6). The pDCs appeared uniquely, whereas mDCs appeared with other T cell subpopulations, such as terminal effector CD4, terminal effector CD8, effector memory CD8, and non-Vd2 gd T cells.

## Discussion

Many recent studies have applied scRNA-seq to examine immune cells in blood or tissues^12,20–25^; however, this technique has not been used to examine their association with gout. In this study, we collected seven blood and three SF samples from patients with acute gout, and five samples from normal controls for scRNA-seq analysis. We found that the length of the clinical course and patient lifestyle may play important roles in the variation in and distribution of immune cell subpopulations. Most blood immune cell subpopulations were similar between gout patients and healthy controls, with only naïve CD4 T cell, classic monocytes, intermediate monocytes, and pDCs being different between subject groups (Fig. 2); the subclusters of these subpopulations also differed. We detected no correlation between subclusters and cell abundance.

It is difficult to differentiate between patients with acute gout and healthy subjects according to blood cell populations. The inflammatory signature of PBMCs has been used to evaluate the role of immune cells in mortality risk associated with COVID-19 inflection^26^, and an energy metabolism-based eight-gene signature has been used to evaluate clinical outcomes of esophageal cancer^27^. To explore differences in the quality and quantity of immune cells between subject groups, we adopted, expanded, and modified inflammatory signature-related gene scores as IS and energy metabolism-related gene scores as ES to evaluate cell subpopulations and subclusters between acute gout and healthy subject groups^17^. The highest IS was found in SF, followed by blood cells from acute gout and healthy control subjects, whereas the opposite results were observed for ES (Fig. 4). These results may provide a new diagnostic marker and therapeutic target for patients with gout.

For patients with acute gout, the timing and duration of gouty arthritis symptoms may play an important role in the variation in and distribution of immune cell subpopulations. Subpopulations of immune cells were similar among patients with similar clinical courses. For example, patients with persistent pain had fewer terminal effector CD4, terminal effector CD8, and non-Vd2 gd T cells but more Th2 cells and non-classic monocytes.

Among cell subpopulations in SF, the most abundant T cell-related subpopulations were Th1, Th1/Th17, effector memory CD8 T, and MAIT cells, whereas macrophages and mDCs were the most common monocytes. These results were inconsistent with the blood sample cell subpopulation results for patients with acute gout. Therefore, we suggest that the modulation of cell migration from blood to SF may play a role in potential gout treatment.

Many studies have shown that immune cells secrete cytokines or chemokines in response to hyperuricemia or urate crystal stimulation; however, there are no data related to interactions among these immune cells^3,6–11,28–33^. In this study, we used the CellChat algorithm to explore cell–cell interactions and found that gout patients and healthy subjects have similar cell–cell interactions, whereas patients with acute gout show more mDC or pDC interactions; these results may be used to develop markers for the prediction and evaluation of therapy effectiveness and clinical courses.

Dynamic changes in immune cell subpopulations and patient lifestyle heavily influence blood immune cells, and acute attacks of gouty arthritis can occur following interactions among these factors and urate crystals, which may explain the complexity of clinical presentations of gouty arthritis, such as asymptomatic hyperuricemia or normal-uricemia gouty attacks. The dynamic changes of subpopulation of immune cells and patient’s real-time lifestyle heavily influence the immune cells in the blood, and the acute attack of gouty arthritis may occur after interaction among these factors and urate crystal. These interactions may explain the complexity of clinical presentations of gouty arthritis, such as asymptomatic hyperuricemia, normal-uricemia gouty attack. We found enriched naïve CD4 T cell and classical monocyte populations in patients with gout, and that the membranous markers of these cells are difficult to block using small molecules or antibodies. We suggest using drugs to modulate the differentiation or function of these cells for the treatment of gout.

## Conclusions

In this study, scRNA-seq analysis of immune cells reveals cellular and molecular insights on inflammatory responses to hyperuricemia or uric crystal and may provide avenues for new diagnostic approaches and therapeutic guidance to improve gout treatment. We also provide two new evaluation systems for exploring inflammation and energy status in patients with gout, for gout patients.

## Supplementary Information

Supplementary Information.

## Data Availability

The single-cell RNA sequencing data for this study were submitted to the NCBI Sequence Read Archive under the BioProject PRJNA861849.

## Abbreviations

SF: Synovial fluid
MSU: Monosodium urate
SU: Serum urate
TNF-α: Tumor necrosis factor-α
IL: Interleukin
scRNA-seq: Single-cell RNA sequencing
ACR/EULAR: American College of Rheumatology/European League Against Rheumatism
UMI: Unique molecular identifiers
PCA: Principal Component Analysis
rPCA: Robust Principal Component Analysis
UMAP: Uniform manifold approximation and projection
SNN: Shared nearest neighbor
IS: Inflammation score
ES: Energy score
TGF-β1: Transforming growth factor-β1
PBMCs: Peripheral blood mononuclear cells
DC: Dendritic cells
NK: Natural killer
MAIT cells: Mucosal associated invariant-T cells
pDCs: Plasmacytoid dendritic cells
mDCs: Myeloid dendritic cells
