# Supplementary material for: Single-cell RNA sequencing of immune cells in patients with acute gout
Source: Sci Rep. 2022 Dec 22;12:22130. doi: 10.1038/s41598-022-25871-2 (PMC9772586; doi:10.1038/s41598-022-25871-2)
Supplement: Supplementary file 1 — Supplementary Information. [file 41598_2022_25871_MOESM1_ESM.docx]

**Table S1.** Clinical information of gouty patients and healthy controls.

|  | **Gender** | **Age (diagnosed)** | **Age (draw blood)** | **Uric acid (mg/dL)** | **Pain period (days)** | **GFR**  **(ml/min/1.73m^2^)** | **Cluster by Richette et al.** |
| --- | --- | --- | --- | --- | --- | --- | --- |
| Case 01 | Male | 30 | 46 | 4.8 | 3 | 74 | C1 |
| Case 02 | Male | 21 | 38 | 8.8 | 3 | 88 | C1 |
| Case 03 | Male | 18 | 43 | 8.8 | 14 | 95 | C2 |
| Case 04 | Male | 39 | 55 | 5.3 | 1 | 92 | C2 |
| Case 05 | Male | 40 | 40 | 6.0 | 1 | 79 | C1 |
| Case 06 | Male | 24 | 29 | 4.4 | 2 | 78 | C2 |
| Case 07 | Male | 33 | 33 | 7.1 | 19 | 106 | C1 |
| Gout 146 | Male | 41 | 49 | 8.9 | 3 | 102 | C1 |
| Gout 151 | Male | 13 | 35 | 7.1 | 2-14 | 102 | C2 |
| Gout 183 | Male | 33 | 43 | 7.8 | 10 | 82 | C2 |
| MH | Male |  | 25 | 7.3 |  | 78 |  |
| RG | Female |  | 37 | 3.9 |  | 116 |  |
| N01F | Male |  | 40 | 5.1 |  | 80 |  |
| N05S | Male |  | 59 | 6.9 |  | 85 |  |
| YB | Male |  | 28 | 7.2 |  | 115 |  |

**GFR: glomerular filtration rate**

**
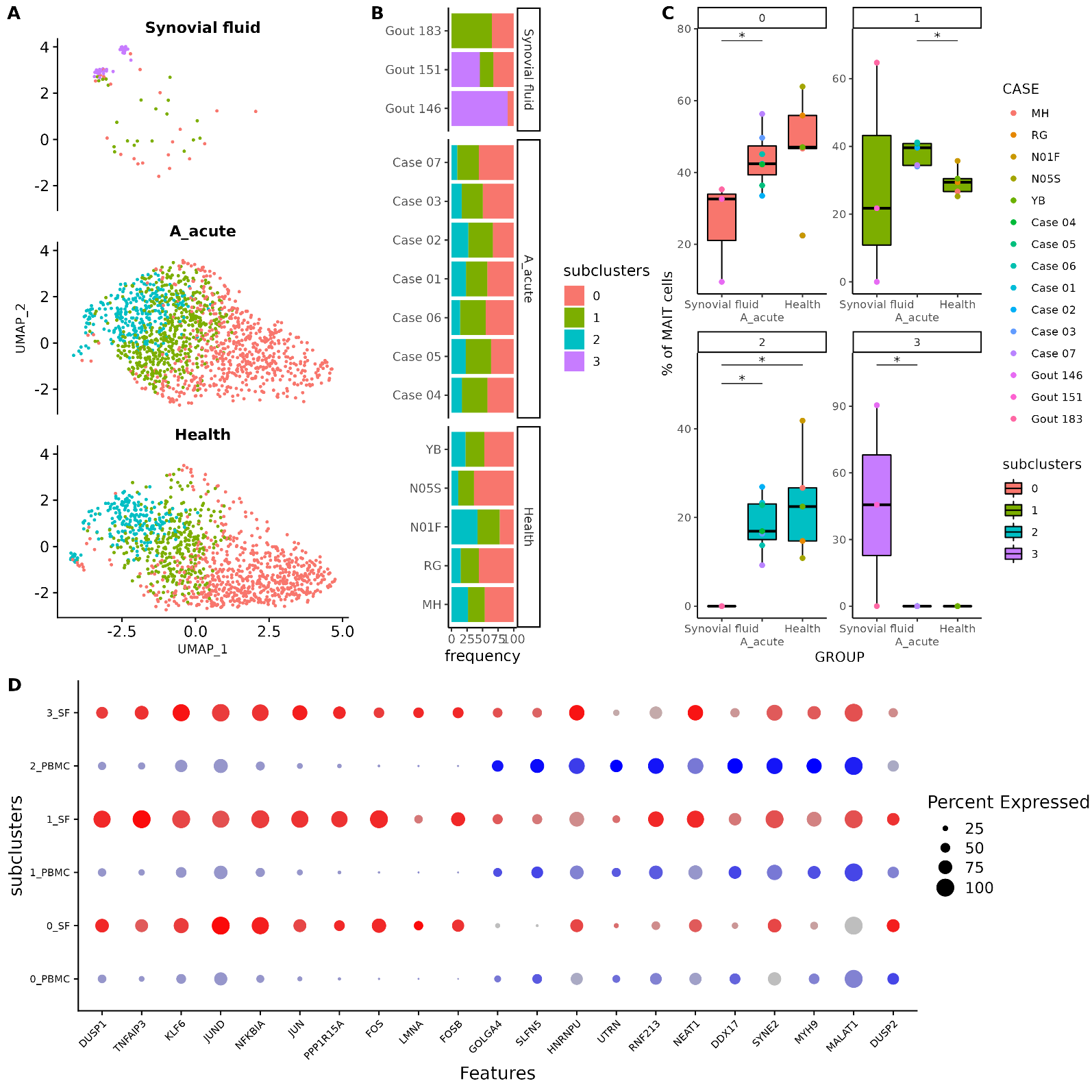
**

**Figure S1.** MAIT cells subclustering results for (A, B) SF and PBMCs from acute gout and healthy subject groups are shown on UMAP coordinates. Stacked bar plot shows relative abundances. (C) Boxplots of subcluster relative abundance between SF samples and PBMCs of acute gout and healthy subject groups (**P* < 0.05). (D) Dot plot of gene expression profiles of the top 10 marker genes in each subcluster.

**
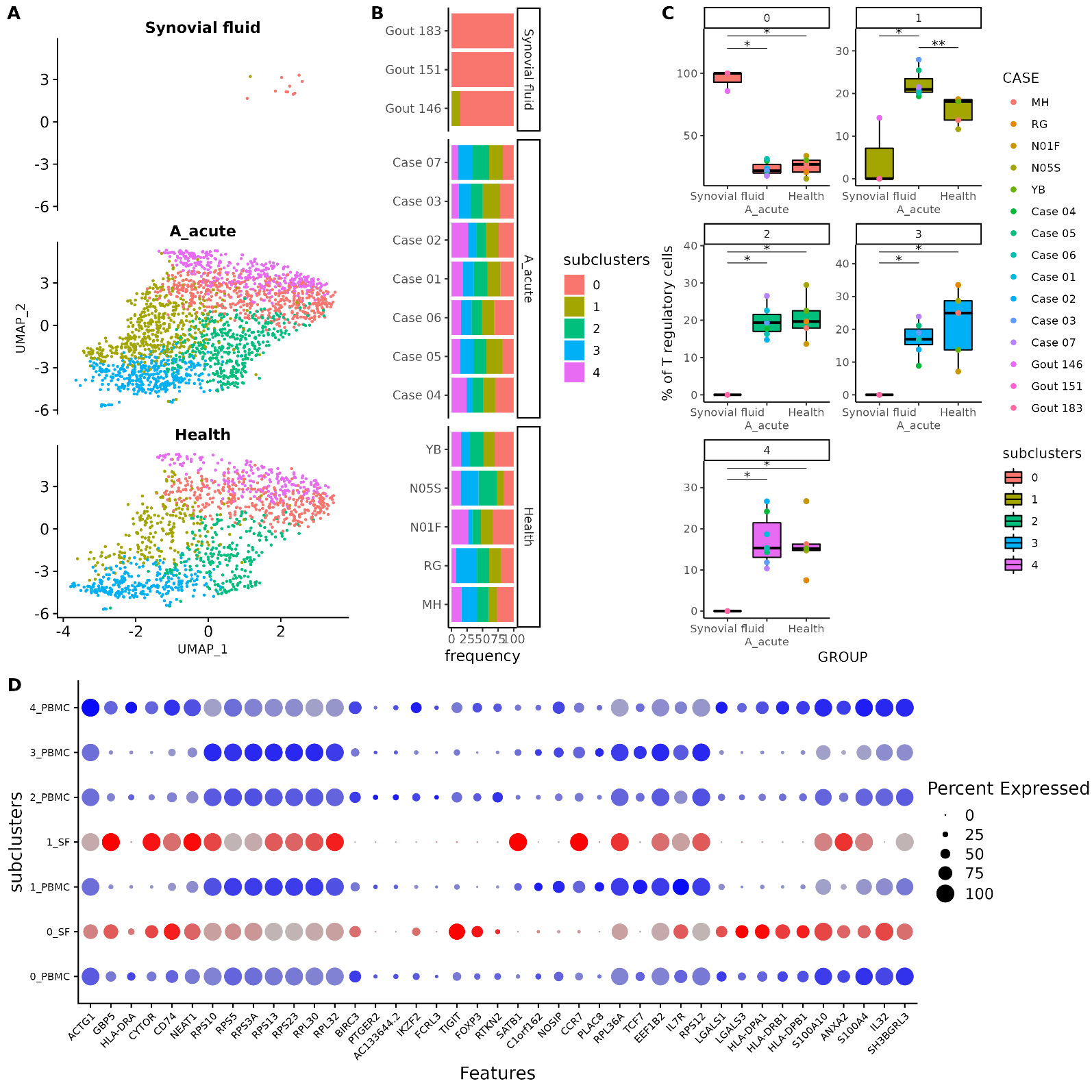
**

**Figure S2.** Regulatory T cells subclustering results for (A, B) SF and PBMCs from acute gout and healthy subject groups are shown on UMAP coordinates. Stacked bar plot shows relative abundances. (C) Boxplots of subcluster relative abundance between SF samples and PBMCs of acute gout and healthy subject groups (**P* < 0.05; ***P* < 0.01). (D) Dot plot of gene expression profiles of the top 10 marker genes in each subcluster.

**
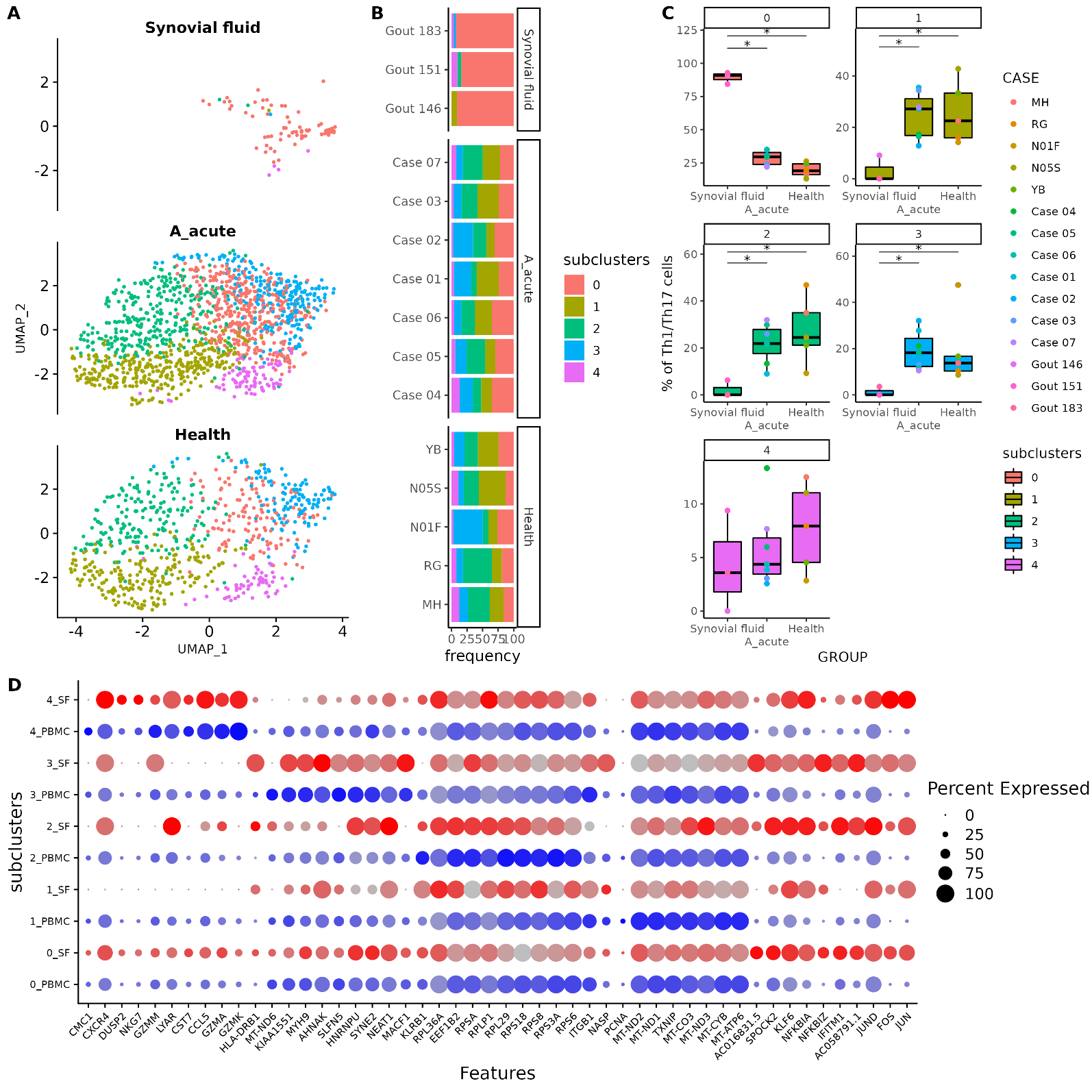
**

**Figure S3.** Th1/Th17 cells subclustering results for (A, B) SF and PBMCs from acute gout and healthy subject groups are shown on UMAP coordinates. Stacked bar plot shows relative abundances. (C) Boxplots of subcluster relative abundance between SF samples and PBMCs of acute gout and healthy subject groups (**P* < 0.05). (D) Dot plot of gene expression profiles of the top 10 marker genes in each subcluster.


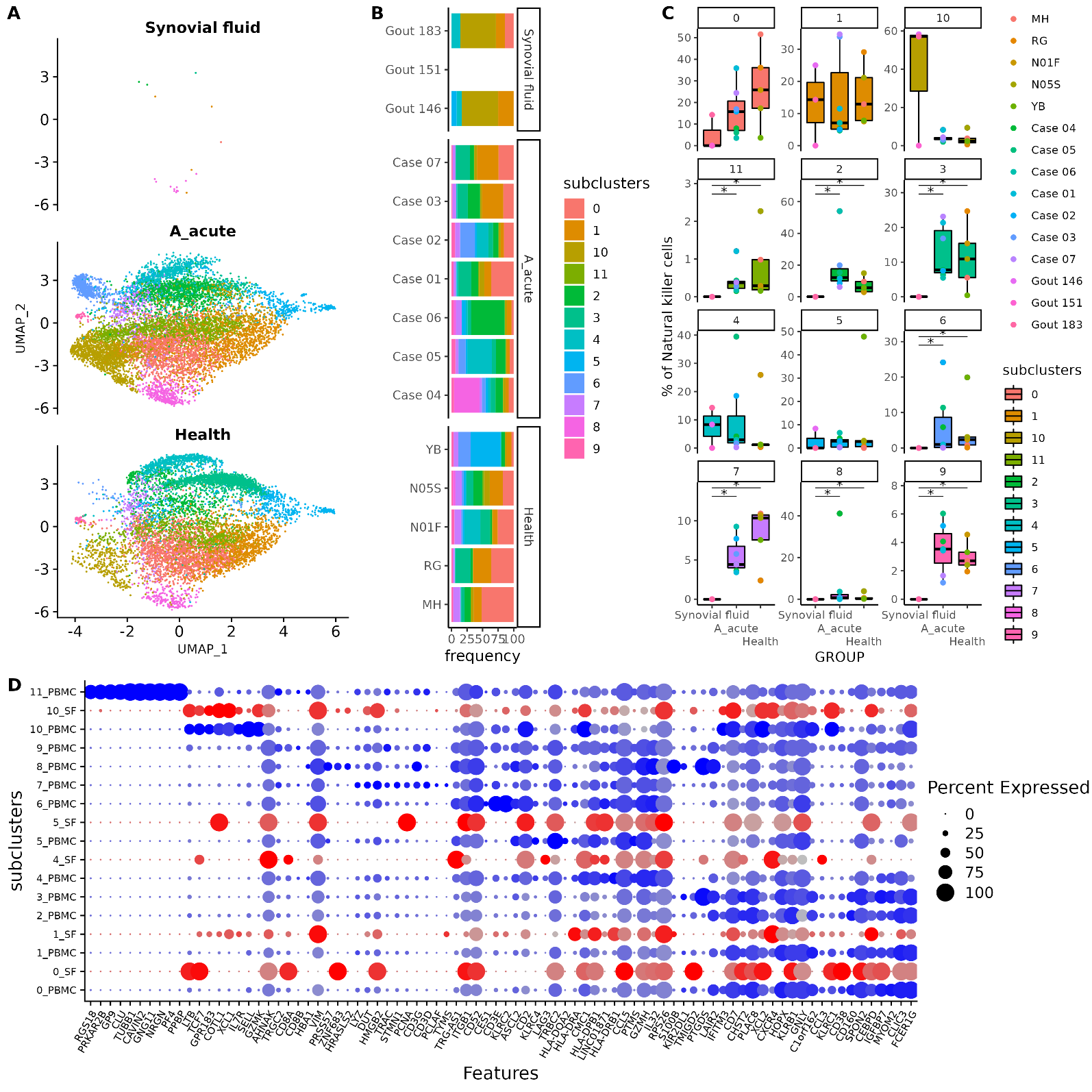


**Figure S4.** Nature killer cells subclustering results for (A, B) SF and PBMCs from acute gout and healthy subject groups are shown on UMAP coordinates. Stacked bar plot shows relative abundances. (C) Boxplots of subcluster relative abundance between SF samples and PBMCs of acute gout and healthy subject groups (**P* < 0.05). (D) Dot plot of gene expression profiles of the top 10 marker genes in each subcluster.


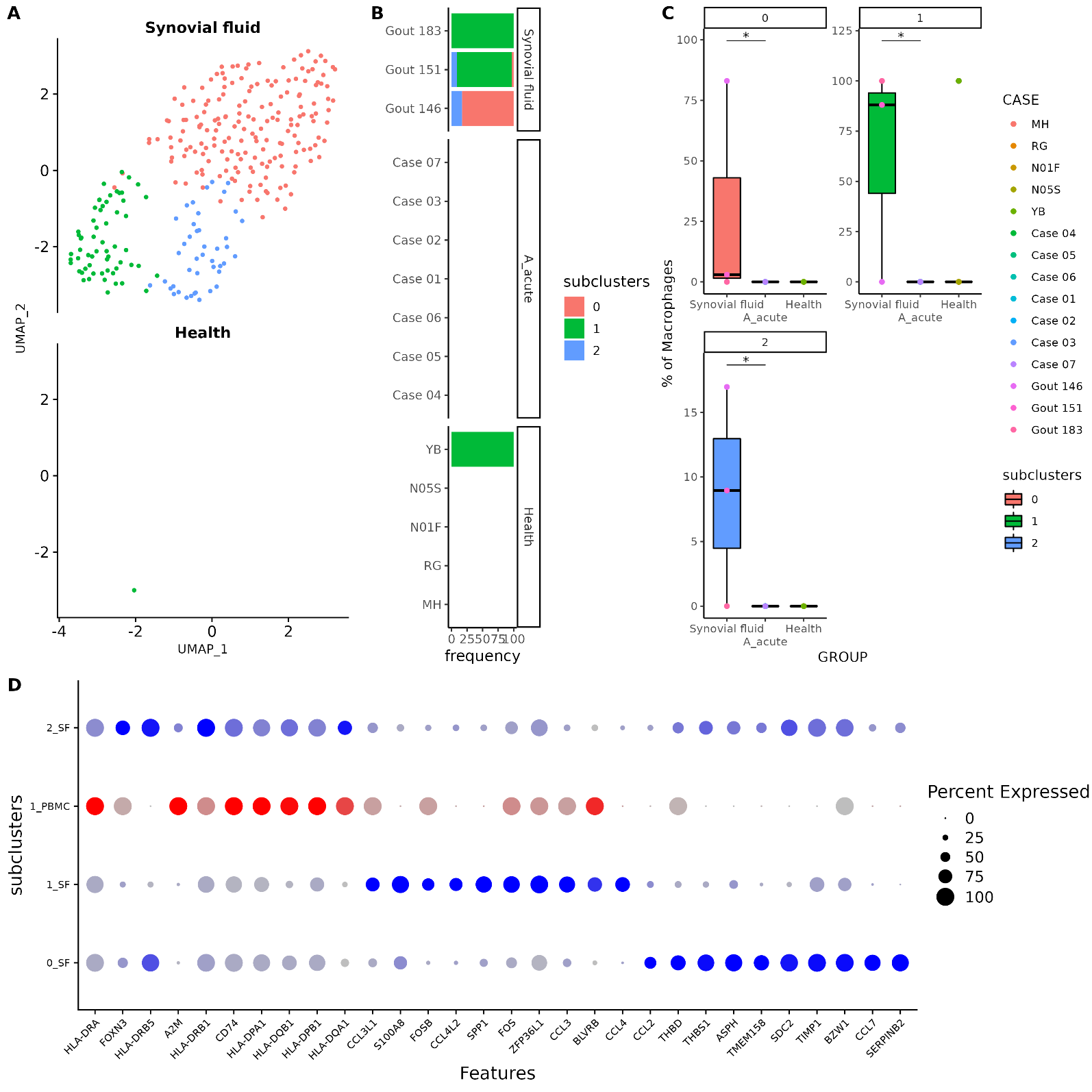


**Figure S5.** Macrophages subclustering results for (A, B) SF and PBMCs from acute gout and healthy subject groups are shown on UMAP coordinates. Stacked bar plot shows relative abundances. (C) Boxplots of subcluster relative abundance between SF samples and PBMCs of acute gout and healthy subject groups (**P* < 0.05). (D) Dot plot of gene expression profiles of the top 10 marker genes in each subcluster.
